# Over-investigation and overtreatment in pediatrics: a survey from the European Academy of Paediatrics and Japan Pediatric Society

**DOI:** 10.3389/fped.2024.1333239

**Published:** 2024-02-22

**Authors:** Lina Jankauskaite, Corinne Wyder, Stefano del Torso, Marina Mamenko, Sandra Trapani, Zachi Grossman, Adamos Hadjipanayis, Karin Geitmann, Hikoro Matsui, Akihiko Saitoh, Tetsuya Isayama, Nora Karara, Alessandra Montemaggi, Farhan Saleem Ud Din, Ketil Størdal

**Affiliations:** ^1^ Department of Paediatrics, Lithuanian University of Health Sciences, Kaunas, Lithuania; ^2^ Institute of Physiology and Pharmacology, Lithuanian University of Health Sciences, Kaunas, Lithuania; ^3^ European Academy of Paediatrics, Brussels, Belgium; ^4^ Paediatric Praxis Kurwerk, Burgdorf, Switzerland; ^5^ ChildCare WorldWide, Padova, Italy; ^6^ Shupyk National Healthcare University of Ukraine, Kiev, Ukraine; ^7^ Ukrainian Academy of Paediatric Specialties, Kyiv, Ukraine; ^8^ Department of Health Sciences, University of Florence, Florence, Italy; ^9^ Paediatric Unit, Meyer Children’s Hospital IRCCS, Florence, Italy; ^10^ Adelson School of Medicine, Ariel University, Ariel, Israel; ^11^ Pediatric Clinic, Maccabi Healthcare Services, Tel Aviv, Israel; ^12^ Medical School, European University Cyprus, Nicosia, Cyprus; ^13^ Primary Care Paediatrician, BVKJ, Hagen, Germany; ^14^ University of Tokyo and School of Medicine, Tokyo, Japan; ^15^ Japan Pediatric Society, Tokyo, Japan; ^16^ Department of Pediatrics, Niigata University Graduate School of Medical and Dental Sciences, Niigata, Japan; ^17^ Division of Neonatology, National Center for Child Health and Development (NCCHD), Tokyo, Japan; ^18^ Paediatric Public Health Office, Berlin, Germany; ^19^ Division of Pediatric and Adolescent Medicine, Oslo University Hospital, Oslo, Norway; ^20^ Department of Pediatric Research, Faculty of Medicine, University of Oslo, Oslo, Norway

**Keywords:** Choosing Wisely, European Academy of Paediatrics, Japan Pediatric Society, child, medical overuse, paediatrics/pediatrics

## Abstract

**Introduction:**

Avoiding over-investigation and overtreatment in health care is a challenge for clinicians across the world, prompting the international Choosing Wisely campaign. Lists of recommendations regarding medical overactivity are helpful tools to guide clinicians and quality improvement initiatives. We aimed to identify the most frequent and important clinical challenges related to pediatric medical overactivity in Europe and Japan. Based on the results, we aim to establish a (European) list of Choosing Wisely recommendations.

**Methods:**

In an online survey, clinicians responsible for child health care in Europe and Japan were invited to rate 18 predefined examples of medical overactivity. This list was compiled by a specific strategic advisory group belonging to the European Academy of Paediatrics (EAP). Participants were asked to rate on a Likert scale (5 as the most frequent/important) according to how frequent these examples were in their working environment, and how important they were considered for change in practice.

**Results:**

Of 2,716 physicians who completed the survey, 93% (*n* = 2,524) came from 17 countries, Japan (*n* = 549) being the largest contributor. Pediatricians or pediatric residents comprised 89%, and 51% had 10–30 years of clinical experience. Cough and cold medicines, and inhaled drugs in bronchiolitis were ranked as the most frequent (3.18 and 3.07 on the Likert scale, respectively), followed by intravenous antibiotics for a predefined duration (3.01), antibiotics in uncomplicated acute otitis media (2.96) and in well-appearing newborns. Regarding importance, the above-mentioned five topics in addition to two other examples of antibiotic overtreatment were among the top 10. Also, IgE tests for food allergies without relevant medical history and acid blockers for infant GER were ranked high.

**Conclusion:**

Overtreatment with antibiotics together with cough/cold medicines and inhaled drugs in bronchiolitis were rated as the most frequent and important examples of overtreatment across countries in Europe and Japan.

## Introduction

“To do no harm” is an overarching ancient principle in medical practice, and still is a valid commitment (1). The increasing availability of tests and treatment procedures in children's health care has the potential to benefit individuals and children's health in general. This increasing access, however, also has the potential to harm if tests and treatments are applied outside their evidence base. Most investigations and treatments have an inherent risk and, thus, need to be balanced against the potential benefits. Over-investigation means a diagnostic work-up that is unlikely to provide useful information to the patient. Overtreatment is a treatment that does not benefit the patient and can be labelled together with over-testing and -diagnosis as “medical overactivity”.

A challenge was raised in an NEJM perspective paper to list diagnostic tests or treatments that were commonly ordered but without any meaningful benefit to most patients (2).

Such lists of clinical recommendations are important tools in the Choosing Wisely (CW) campaign to communicate with clinicians for translation into clinical care (3). These recommendations are intended to spur conversations among clinicians and patients about the overuse of medical tests and procedures that provide little benefit and, in some cases, may harm (4). Recommendations should cover tests and procedures commonly used, not supported by evidence, and expose patients to unnecessary risk (5). Short statements of 1–2 sentences are supported by a paragraph summarizing the evidence and with relevant references from the literature. These recommendations do not replace guidelines or systematic reviews but work as a communication tool to convey well-established evidence where practice needs to change.

There is currently a wide selection of clinical recommendations concerning child health from numerous national societies in Europe, Australia, and North America, most of which are collected on the website of the European Academy of Pediatrics (EAP) (6). Among these national recommendations, there are some similarities and recurring topics, whereas others are relevant to specific working environments and countries.

Recently, we conducted a survey among clinicians from five European countries regarding extent of medical overactivity and perceived drivers (7). In this survey, the medical overactivity was established as a frequent problem (81% of responders). Perceived expectations from family and patients were the most significant driver for medical overactivity in the participating countries. However, this first survey did not intend to identify common specific topics of medical overactivity (7).

The present survey aimed to identify the most frequent and important clinical challenges related to medical overactivity that European and Japanese clinicians meet in their working environments. Further, we explored similarities and differences between countries.

## Materials and methods

### Survey creation and distribution

The EAP “CW” strategic advisory group engaged in an internal discussion and research on data from international CW initiatives to identify topics related to over-investigation and overtreatment. The 18 topics considered the most relevant are listed in Table 1. The goal was then to gather information from clinicians on the frequency and importance of these examples of overtreatment and over-testing experienced in pediatric care. In the introductory section of the survey, overtreatment was defined as treatment that does not benefit and may even harm the patient. Over-investigation was defined as a diagnostic work-up or referral that is unlikely to provide relevant information to the patient. Before starting the study, the survey was piloted by a survey committee which consisted of members from EAP.

**Table 1:** Response alternatives in the survey of most frequent and important topics of medical overactivity.

Q1	….acid blockers or motility agents used for the treatment of gastroesophageal reflux in infants
Q2	….cough and cold medicines prescribed, recommended or used for respiratory illnesses in young children?
Q3	….routine treatment of acute otitis media (AOM) with antibiotics in children (>6 m of age)?
Q4	….routinely use steroids or bronchodilators in infants with bronchiolitis?
Q5	….routine chest-x-rays (ordered/undertaken) for the diagnosis of bronchiolitis in children?
Q6	….routinely use antibiotics in newborns (more than 36–48 h of age) when bacterial infection is unlikely?
Q7	….blood exams prescribed/ordered in children with acute pharyngitis?
Q8	….urine samples taken/collected from children >2 months of age with symptoms and signs of respiratory infection, (except when the child is septic, predisposed to urinary tract infection or has additional specific urinary tract symptoms)?
Q9	….IV fluids given to children with mild to moderate dehydration, before a trial of oral fluids?
Q10	….screening panels (IgE tests) prescribed/recommended/performed for food allergies without previous consideration of the pertinent/relevant medical history?
Q11	….order routine chest-x-rays for the diagnosis of asthma in children?
Q12	….routine electroencephalogram or studies of neuroimaging (CT, MRI) performed in children with simple febrile convulsion?
Q13	….routinely check vitamin D level in healthy children?
Q14	….IV antibiotics prescribed for a predetermined time duration for patients hospitalized with infections such as pyelonephritis, osteomyelitis, and complicated pneumonia without considering early transition to oral antibiotics?
Q15	….hospitalization continued in well-appearing febrile infants once the results of bacterial cultures (i.e., blood, cerebrospinal, and/or urine) have been confirmed to be negative for 24–36 h, and adequate outpatient follow-up can be ensured/provided?
Q16	….phototherapy initiated in term or late preterm well-appearing infants with neonatal hyperbilirubinemia if their bilirubin levels are below the level at which the AAP guidelines would recommend treatment?
Q17	….broad-spectrum antibiotics, such as ceftriaxone, used for children hospitalized with uncomplicated community acquired pneumonia (CAP) instead of using narrow-spectrum antibiotics, such as penicillin, ampicillin, or amoxicillin?
Q18	….start IV antibiotic therapy on well-appearing newborn infants with isolated risk factors for sepsis (such as maternal chorioamnionitis, prolonged rupture of membranes, or untreated group B streptococcal colonization) instead of using clinical tools (such as an evidence-based sepsis-risk calculator) to guide management?

### Data collection

The survey was created on the research platform used by the European Academy of Paediatrics Research in Ambulatory Settings network (EAPRASnet). A web link was distributed to the network members of EAPRASnet and national pediatric representatives of the EAP urging them to invite members in their national societies and Young EAP representatives to participate. The Japan Pediatric Society (JPS) also distributed the survey in their country, accordingly.

Once the survey was distributed, data were collected for six weeks starting 19th September 2022, in EAPRASnet countries and 11th November 2022, in JPS. Two reminders were sent out during the data collection period. Baseline characteristics, such as country, gender, age group, experience, working environment, and career stage were collected in addition to the grading of clinical examples, in terms of frequency and importance. The 5-point Likert scale was used to evaluate all the selected topics, and responders were asked to score “how frequently this practice is seen” and “how important it is to change this practice” in their working environment.

Only the data from fully completed surveys were considered for further analysis. The answers were collected anonymously; thus, no Ethical consent was required.

### Statistical data analysis

The collected data were stored in Microsoft Excel and analyzed using IBM SPSS Statistics version 29.0 software (SPSS Inc., Chicago, IL, USA) for Windows. Data are presented as count and a percentage (%). Continuous variables were expressed as mean +/− standard deviation (SD); ordinal variables were expressed as median. Ordinal variables of the two groups were compared by the Mann–Whitney *U*-test.

Descriptive analyses were performed only for countries with more than 30 respondents, whereas those with fewer than 30 respondents were pooled into the category “others”. For the countries with >100 respondents the top ten highest-scored topics were analyzed and depicted on heat map. For comparison between the working settings, “university hospital” and “tertiary/secondary hospital” were pooled and compared with “primary pediatrics”. Responses from GPs and non-pediatric specialists were excluded from this analysis.

## Results

In total, 2,716 fully completed survey responses were collected and analysed. Eight countries had more than 100 respondents, and a further nine countries had 30–100 respondents (Table 2).

**Table 2:** Number of respondents who completed the survey by country (*n* = 2,716).

Country	*n*
Japan	549
Italy	268
Spain	230
Switzerland	206
Germany	194
Ukraine	193
Norway	188
Belgium	136
France	87
Lithuania	78
Denmark and Greenland	77
Slovenia	74
Poland	62
Austria	55
Estonia	43
Israel	41
Croatia	30
Others (<30 respondents per country)*	205

* Other participating countries: Portugal (*n* = 26), Netherlands (*n* = 23), Hungary (*n* = 19), Turkey (*n* = 16), Belarus and Ireland (*n* = 14), Finland and Malta (*n* = 12), Luxembourg and UK (*n* = 10), Cyprus (*n* = 9), Andorra (*n* = 7), Sweden (*n* = 6), Bulgaria and Greece (*n* = 5), Czech republic (*n* = 3), Georgia (*n* = 2) and Angola, Armenia, Bermuda, Burundi, Canada, Haiti, North Macedonia, Montenegro, Romania, Slovakia, South Sudan and USA (*n* = 1).

In general, 61.0% of responders (*n* = 1,656) were female. Most participants were between 31 and 60 years of age and had 10–30 years of working experience (Table 3). Participants from secondary/tertiary care or university hospitals comprised more than 50%. Approximately 40% of respondents were primary care pediatricians (PCPs), and only 7.3% were general practitioners (GPs) or adult subspecialists (Table 3). The majority of participants were pediatricians or pediatric subspecialists (*n* = 2,173, 80%).

**Table 3:** Characteristics of the respondents in the survey of medical overactivity.

	Total *n* = 2,716
Gender (*n*, %)	
Female	1,656 (61.0)
Age group (*n*, %)	
≤30 y	190 (7.0)
31–40 y	690 (25.4)
41–50 y	673 (24.8)
51–60 y	653 (24.0)
≥60 y	510 (18.8)
Experience (*n*, %)	
<10 y	648 (23.9)
10–30 y	1,394 (51.3)
>30 y	674 (24.8)
Working environment (*n*, %)	
GP or adult subspecialist	199 (7.3)
Primary care pediatrics	1,064 (39.2)
Secondary/tertiary care hospital	767 (28.2)
University hospital	686 (25.3)
Career stage (*n*, %)	
Pediatrician or pediatric subspecialist	2,173 (80.0)
GP or adult subspecialist	309 (11.4)
Resident	234 (8.6)

*n*, total number; y, years; GP, general practitioner.

**Figure 1:**
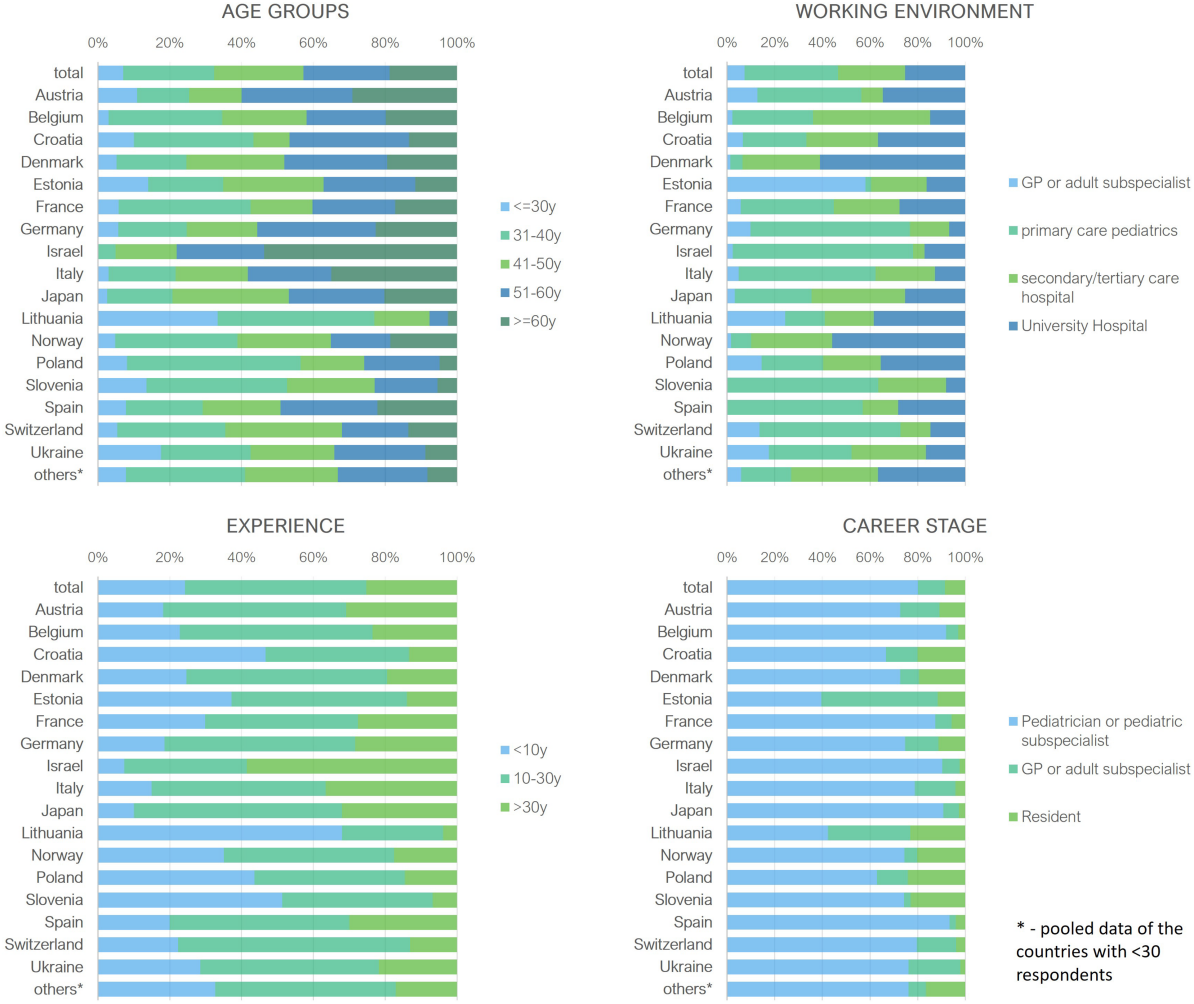
Baseline characteristics of participants from countries with ≥30 respondents. y, years; GP, general practitioners.

In the 17 countries with more than 30 responders, PCPs constituted the majority, though there were a few exceptions. In Estonia, answers from GPs dominated the responses, while in Denmark and Norway, most participants were university hospital physicians (Figure 1). Norway, Denmark, and Lithuania had the smallest number of PCPs who responded, whereas responses from Germany, Israel, Italy, Slovenia, Spain, and Switzerland were predominantly from PCPs. A higher number of less experienced participants responded from Lithuania, Croatia, Poland, and Slovenia (Figure 1).

### All participants: statements about overinvestigation and overtreatment

**Figure 2:**
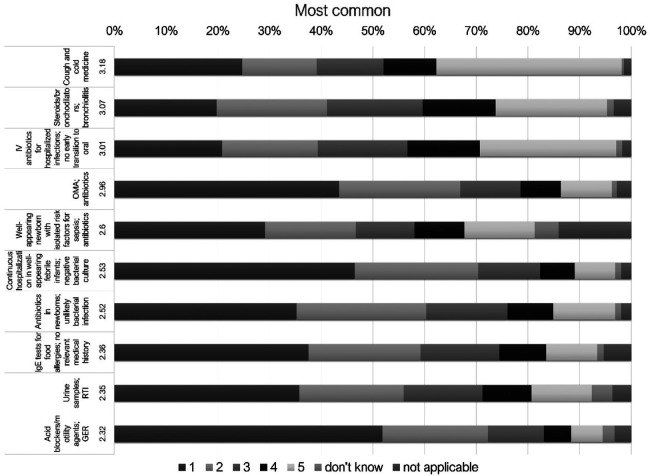
10 most *frequent* statements about overinvestigation and overtreatment ranked in Likert scale for all participants. IV, intravenous; SBI, serious bacterial infection; AOM, acute otitis media; CAP, community acquired pneumonia; IgE, immunoglobulin E; RTI, respiratory tract infections; GER, gastroesophageal reflux.

Overall, the highest rated statements regarding frequency were as follows: cough and cold medicine (Q2, average score 3.18 on the Likert scale), steroids/bronchodilators use for bronchiolitis (Q4, 3.07), intravenous (IV) antibiotic prescription for a predetermined time duration for hospitalized pediatric patients with infections without early transition to oral antibiotics (Q14, 3.01), and antibiotic use in acute otitis media (AOM) (Q3, 2.96) (Figure 2). Acid blocker/motility agents for infant gastroesophageal reflux (GER) (Q1), urine samples in respiratory tract infections (RTIs) (Q8), IgE tests for food allergies without relevant medical history (Q10), prolonged antibiotic use in newborns (Q6), continuous hospitalization ininfants after negative bacterial culture results (Q15), and IV antibiotic therapy in well-appearing newborn with isolated risk for sepsis (Q18) were other items which ranked among the top 10 topics.

**Figure 3:**
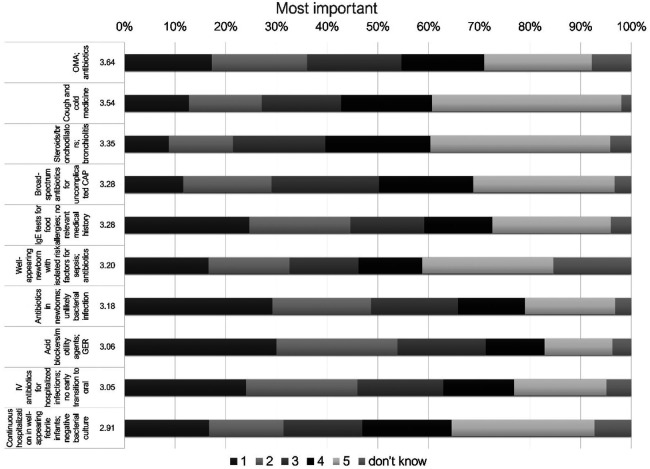
10 most *important* statements about overinvestigation and overtreatment ranked in Likert scale for all participants. IV, intravenous; SBI, serious bacterial infection; AOM, acute otitis media; CAP, community acquired pneumonia; IgE, immunoglobulin E; RTI, respiratory tract infections; GER, gastroesophageal reflux.

Interestingly, the topics scoring high on the frequency of use were also the topics ranked as most important for change (Figure 3): antibiotics for AOM and cough medicine, as well as steroids/bronchodilators in bronchiolitis were rated highest (an average score of 3.64, 3.54, and 3.35, respectively).

### Comparing hospital and primary pediatric settings

**Figure 4:**
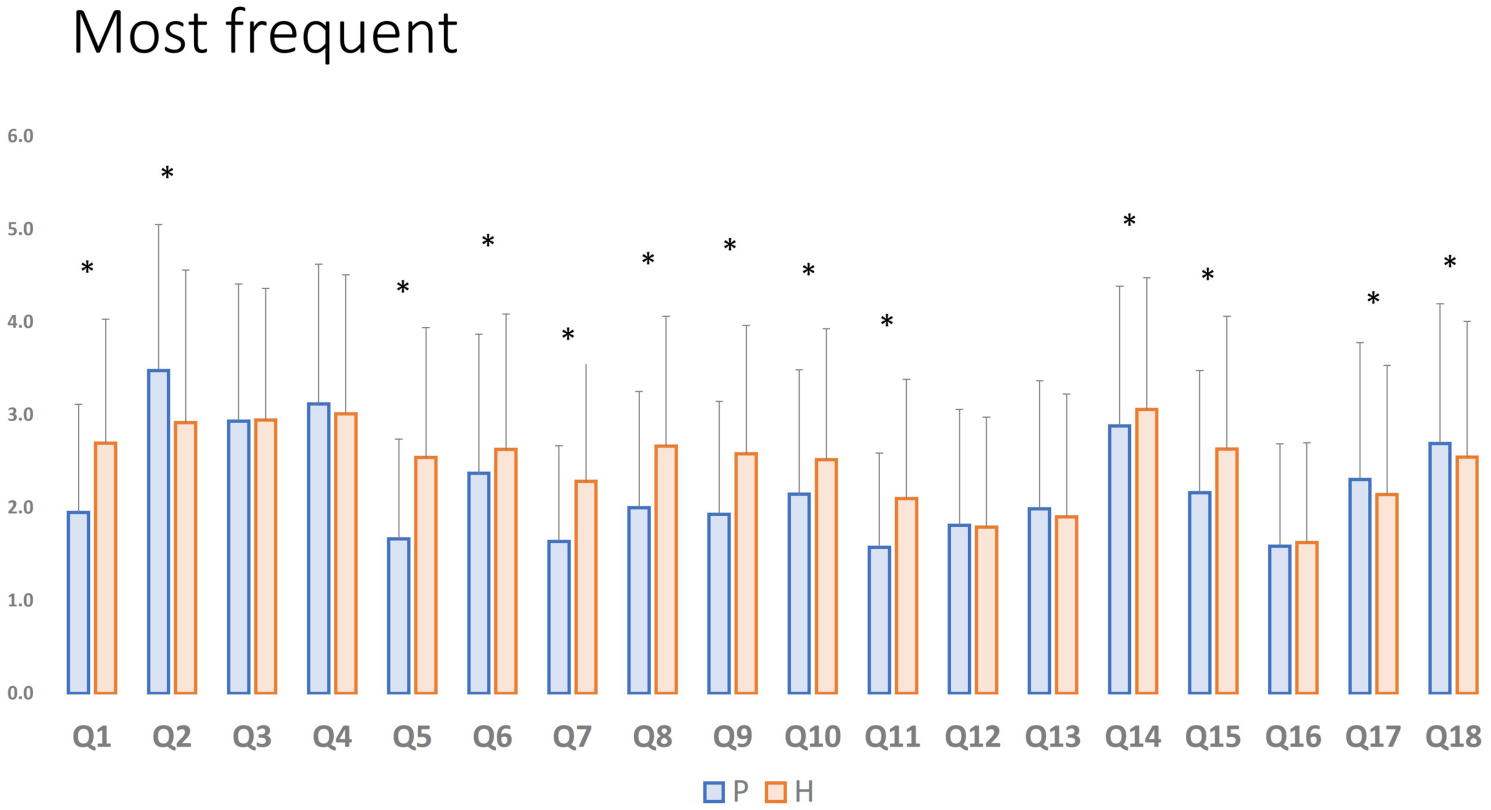
Differences between the ranking for medical overactivity by *frequency* in hospital and primary pediatric settings. P, primary pediatrics; H, hospital settings; Q, question; *Significant difference, where *p* < 0,05.

**Figure 5:**
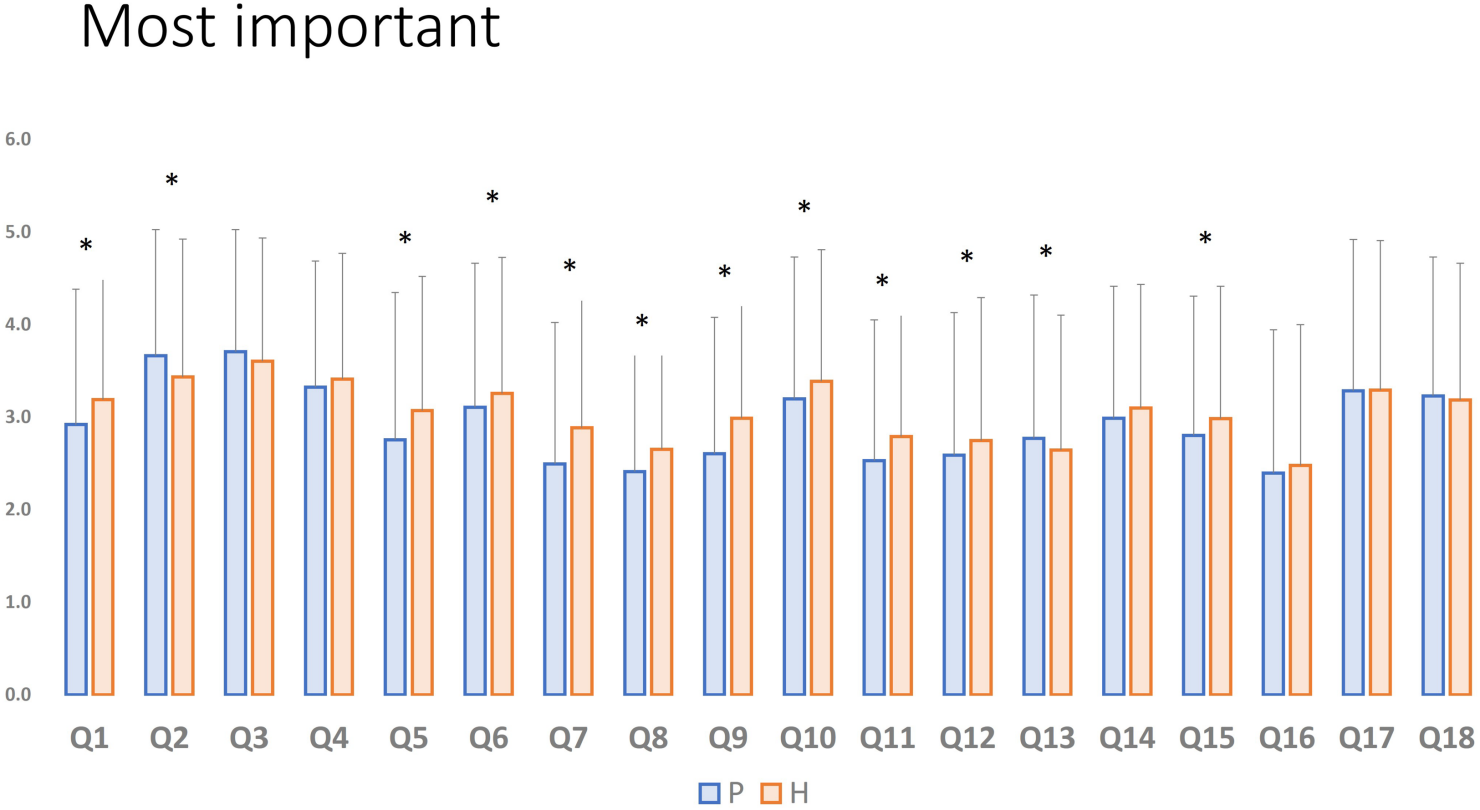
Differences between the ranking for medical overactivity by *importance* in hospital and primary pediatric settings. P, primary pediatrics; H, hospital settings; Q, question; *Significant difference, where *p* < 0,05.

In total, we received 1,064 (42.3%) responses from primary pediatricians and 1,453 (57.7%) from hospital settings (Table 4). Participants from both settings agreed on the majority of the top ten topics, however, with some differences. Cough and cold medicine, routine use of steroids and bronchodilators for bronchiolitis and antibiotic use in AOM were the most frequent topics in respondents from primary pediatrics. Bronchiolitis drugs and antibiotics in AOM were ranked highest together with predetermined IV antibiotic duration in hospitalized patients in respondents from the hospital settings (Table 5). Responses for all the 18 topics for primary care and hospital settings are shown in Figure 4 (frequency) and Figure 5 (importance).

**Table 4:** Distribution of participants between primary and hospital care by country.

	P (*n*, %)	H (*n*, %)
Total (from all the countries)	1,064 (42.3)	1,453 (57.7)
Belgium	46 (34.6)	87 (65.4)
Germany	130 (74.3)	45 (25.7)
Italy	154 (60.4)	101 (39.6)
Japan	177 (33.3)	354 (66.7)
Norway	16 (8.6)	169 (91.4)
Spain	131 (57.0)	99 (43.0)
Switzerland	122 (68.5)	56 (31.5)
Ukraine	67 (42.1)	92 (57.9)
Other countries	221 (32.9)	450 (67.1)

P, primary pediatrics; H, hospital settings; n, number.

The survey answers from general practice respondents were excluded.

**Table 5:** Ranking of all the topics for medical overactivity by *frequency* and *importance* in different settings.

Importance		Q1	Q2	Q3	Q4	Q5	Q6	Q7	Q8	Q9	Q10	Q11	Q12	Q13	Q14	Q15	Q16	Q17	Q18
	P	2.92	3.66	3.71	3.32	2.75	3.11	2.49	2.41	2.60	3.20	2.53	2.59	2.77	2.99	2.80	2.40	3.29	3.23
	H	3.19	3.44	3.61	3.41	3.07	3.26	2.89	2.65	2.99	3.39	2.79	2.75	2.65	3.10	2.98	2.48	3.29	3.19
Frequencies		Q1	Q2	Q3	Q4	Q5	Q6	Q7	Q8	Q9	Q10	Q11	Q12	Q13	Q14	Q15	Q16	Q17	Q18
	P	1.95	3.48	2.93	3.12	1.66	2.37	1.64	2.00	1.93	2.14	1.57	1.81	1.99	2.88	2.16	1.59	2.30	2.69
	H	2.70	2.92	2.94	3.01	2.54	2.63	2.28	2.66	2.58	2.52	2.10	1.79	1.90	3.06	2.63	1.62	2.14	2.54

P, primary pediatrics; H, hospital settings; Q, survey question.

Data is represented as a heatmap where red are the highest and white the values less than 2 of the average according to Likert scale.

### Comparing countries with >100 participants

Upon further analysis regarding *frequency of use* in the countries with more than 100 respondents, we found that three topics were among the top ten for all the countries: inhaled drugs in bronchiolitis, predetermined IV antibiotic duration, and antibiotics in well-appearing newborns with isolated risk factors (Table 6). However, we noted some variations among countries: cough and cold medications and routine antibiotics for AOM were among the top ten for all countries except for Norway, and routine urine samples in RTIs were not in the top ten only for Ukraine. Japan, contributing with more than twice as many respondents as any other country, gave high scores for chest x-rays in bronchiolitis and IV fluids in mild/moderate dehydration.

**Table 6:** Highest ranked topics for medical overactivity by *frequency* in eight countries with >100 respondents.

Frequency	Belgium	Germany	Italy	Japan	Norway	Spain	Switzerland	Ukraine	Total
Q1	3.24	1.92	2.08	2.59	2.40	2.65	1.82	2.23	**2** **.** **34**
Q2	2.96	3.64	3.84	3.92	1.55	2.59	3.22	3.25	**3** **.** **23**
Q3	2.84	2.27	3.49	2.34	1.95	3.50	2.38	3.75	**2** **.** **90**
Q4	3.28	3.37	3.79	2.77	2.08	2.96	2.19	3.72	**3** **.** **04**
Q5	1.84	1.69	1.88	2.43	2.40	1.59	1.38	3.45	**2** **.** **13**
Q6	2.32	2.64	3.08	2.26	2.04	2.55	2.36	3.15	**2** **.** **49**
Q7	1.44	1.55	1.49	1.97	3.15	1.26	1.74	2.72	**2** **.** **04**
Q8	2.42	2.30	2.34	2.40	2.29	2.25	1.99	2.64	**2** **.** **35**
Q9	2.54	2.31	2.12	2.73	1.86	1.86	1.68	2.44	**2** **.** **28**
Q10	2.39	2.53	2.45	1.92	3.02	2.23	1.79	2.82	**2** **.** **34**
Q11	1.73	1.63	1.52	1.78	1.96	1.64	1.55	2.38	**1** **.** **85**
Q13	2.32	2.22	1.98	1.43	1.53	1.46	1.31	3.05	**1** **.** **81**
Q14	2.46	2.02	2.28	1.12	2.59	1.95	2.31	1.91	**1** **.** **94**
Q15	2.89	3.18	3.41	2.99	2.57	2.65	2.53	3.84	**3** **.** **02**
Q16	2.07	2.23	2.71	2.88	1.70	2.25	1.77	3.42	**2** **.** **45**
Q17	1.71	1.43	1.61	1.68	1.21	1.33	1.27	2.84	**1** **.** **63**
Q18	1.48	2.09	3.11	2.12	1.38	1.88	1.54	4.16	**2** **.** **22**
Q19	2.48	2.59	3.00	2.57	1.99	2.32	2.19	3.51	**2** **.** **58**

Q-survey question. Data is represented as a heatmap where red are the highest and white the values less than 2 of the average according to Likert scale.

**Figure 6:**
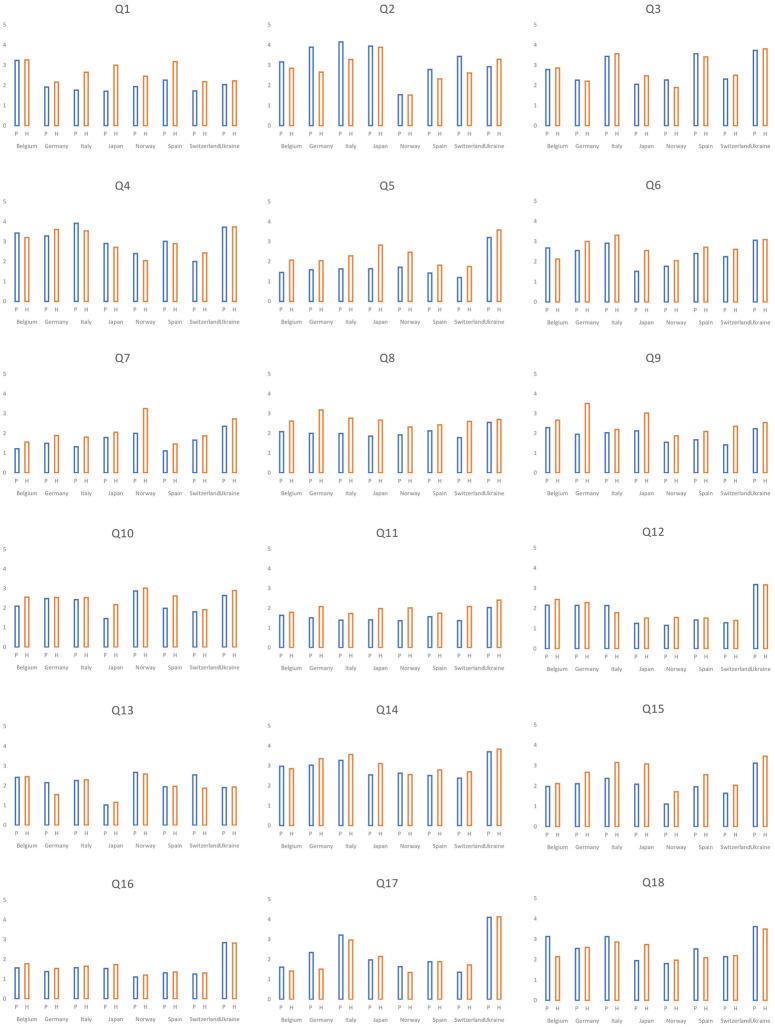
Distribution of the answers as for *frequency* in eight countries with >100 respondents according to work settings. P, primary pediatrics; H, hospital settings; Q, question.

Comparing countries with >100 participants, most participants from Belgium, Japan and Norway were practitioners working in hospital settings, while the majority of respondents from Germany, Italy, and Spain were PCPs (Figure 1). Figure 6 provides the responses for each country split into primary and hospital care. For example, cough medicine scored high in primary pediatrics in the majority of countries.

The analysis regarding the *importance for change of practice* in the countries with more than 100 respondents showed that two out of the top 10 topics were common for all: inhaled drugs in bronchiolitis and routine use of antibiotics for AOM (Table 7). Also, among the top ten most important topics were predetermined IV antibiotic duration (all except Japan). Drugs prescription in infant GER was considered among the five most important in Belgium, Italy, Spain, Switzerland, and Norway.

**Table 7:** Highest ranked topics for medical overactivity by *importance* in eight countries with >100 respondents**.**

Importance	Belgium	Germany	Italy	Japan	Norway	Spain	Switzerland	Ukraine	Total
Q1	3.79	2.41	3.32	2.43	3.53	3.09	2.99	3.59	**3** **.** **04**
Q2	3.26	3.58	3.77	3.34	2.88	3.82	3.90	4.38	**3** **.** **53**
Q3	3.30	3.53	3.48	3.81	3.16	3.84	3.48	4.29	**3** **.** **66**
Q4	3.06	2.69	3.52	3.45	2.94	3.57	3.03	4.27	**3** **.** **33**
Q5	2.76	2.25	2.50	3.24	2.98	2.78	2.58	4.10	**2** **.** **91**
Q6	2.57	3.07	3.22	3.44	3.13	2.98	2.94	4.19	**3** **.** **19**
Q7	2.24	2.06	2.08	3.11	3.30	2.18	2.57	3.61	**2** **.** **71**
Q8	2.25	1.99	2.09	2.63	2.71	2.65	2.44	3.37	**2** **.** **51**
Q9	2.78	2.46	2.30	3.08	2.74	2.43	2.67	3.82	**2** **.** **81**
Q10	2.87	3.24	2.92	3.90	3.79	2.85	2.80	3.89	**3** **.** **31**
Q11	2.50	2.09	2.18	3.20	2.57	2.45	2.48	3.60	**2** **.** **67**
Q12	2.67	2.24	2.63	2.49	3.01	3.00	2.48	3.83	**2** **.** **70**
Q13	2.52	2.31	2.36	3.50	2.38	2.13	2.16	3.70	**2** **.** **68**
Q14	2.09	2.87	2.51	3.08	2.67	2.46	3.06	3.03	**2** **.** **72**
Q15	2.62	2.91	2.95	3.12	2.88	3.00	2.94	4.22	**3** **.** **06**
Q16	2.71	2.51	2.84	3.23	2.44	2.70	2.63	4.19	**2** **.** **91**
Q17	2.14	1.74	2.02	3.21	1.91	2.13	2.12	3.70	**2** **.** **46**
Q18	2.59	3.05	3.49	3.97	2.62	2.76	2.71	4.60	**3** **.** **30**
Q19	2.89	2.83	3.13	3.42	3.02	2.96	2.89	4.41	**3** **.** **19**

Q-survey question. Data is represented as a heatmap where red are the highest and white the values less than 2 of the average according to Likert scale.

Five topics in our survey covered antibiotic use and its duration in different clinical situations. Including all respondents, the five antibiotic topics ranked among the 10 highest regarding both frequency and importance; the only discrepancy was the frequency of broad-spectrum antibiotics for community-acquired pneumonia. All five antibiotic topics were included among the top 10 most important in Italy, Spain, Switzerland, Germany, and Ukraine. Respondents from Belgium considered only three topics related to antibiotics as highly important (Table 7).

**Figure 7:**
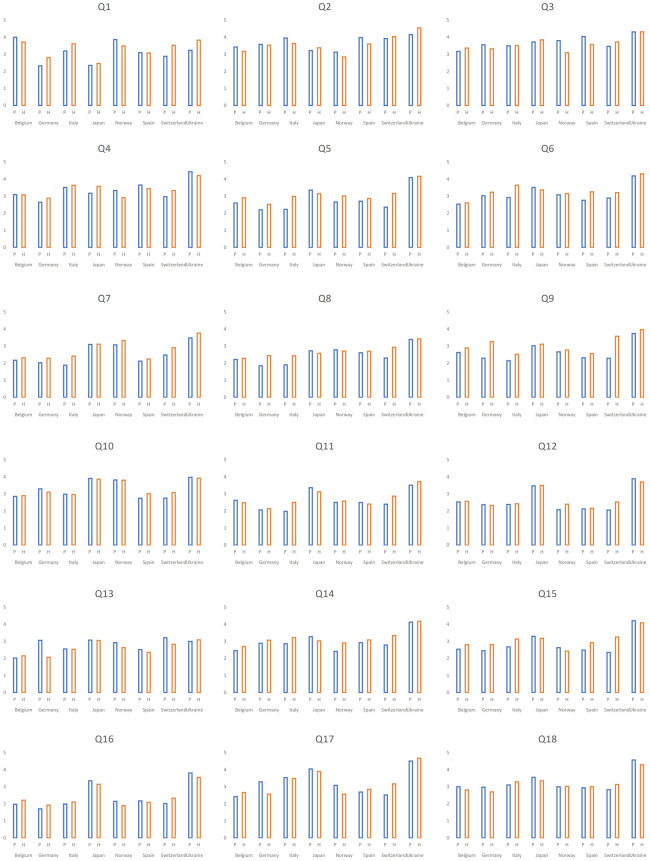
Distribution of the answers as for *importance* in eight countries with >100 respondents according to work settings. P, primary pediatrics; H, hospital settings; Q, question.

Overall, we observed that some of the countries responded with lower values on the Likert scale compared to others (Figures 6, 7). Indeed, regarding the frequency, Switzerland and Norway demonstrated the lowest scored results (the majority scored lower than 2), and only one topic was ranked higher than 3 from all the proposed topics in Switzerland, and 2 in Norway. In contrast, ten topics scored >3 among Ukrainian respondents (Table 6).

## Discussion

In this survey covering EAP countries and Japan, we found striking similarities across countries in the perception of the most frequent topics for medical overactivity. Overuse of antibiotics in different clinical scenarios was regarded as frequent and important in all countries. Supportive therapy which is frequently used in airway infections, such as cough/cold medicines and inhaled steroids and bronchodilators, was also ranked high among practitioners as an example of overtreatment.

Young children are frequently prescribed antibiotics. However, most healthcare contacts due to febrile episodes in this age group are self-limiting viral or bacterial infections, and most of them do not need antibiotics (8). Globally, the consumption of antibiotics in children <5 years for lower RTIs increased by 43% from 2000 to 2018, though antibiotic use in high-income countries appears to be stable over time (9). The increase in antimicrobial resistance has prompted an action plan by the WHO to reduce the current overuse of antibiotics (10). To counteract further increase in antimicrobial resistance, limiting unnecessary prescribing of antibiotics and antibiotic stewardship should be the main priority.

The participating countries in this survey display a wide variation in the current use of antibiotics against childhood lower airway infections, ranging from the lowest in the Netherlands and Scandinavia to countries in Southern Europe with the highest antibiotic use (9). Also, for AOM and upper RTIs, there is a wide variation in antibiotic use with high consumption in Italy compared to the Netherlands and Switzerland (11). In our survey, respondents from countries with the most restrictive actual use of antibiotics (Norway and Switzerland) gave overall lower scores for topics covering antibiotic use compared to Italy and Spain. The differing responses in our survey suggest that clinicians perceive and are conscious of whether the antibiotic prescription in their working environment is in line with global aims to restrict unwarranted use.

Airway infection is the main reason for unscheduled contact with primary care for children and causes a large proportion of referrals and hospital admissions. Because self-limiting viral or bacterial infections predominate, supportive therapy is the mainstay of therapy. However, antitussive and mucolytic medications are not recommended due to their unproven efficacy and risk of toxicity (12, 13). Further, routine bronchodilators were not superior to saline in a randomized trial (14), and are not recommended for routine use in updated international guidelines (15, 16). The discomfort among clinicians to “do something” when an infection is not suited for antimicrobial therapies, may drive the overprescription of supportive drugs.

Overuse of cough and cold medications was overall ranked high (overall #1 in frequency and #2 in importance). However, in some countries such as Norway, this was not perceived either as frequent or important. Most respondents in Norway worked in hospital environments, and PCPs and family doctors are likely to meet the expectations from caregivers regarding non-severe respiratory tract infections more frequently than clinicians in secondary care. We are not aware of data comparing the actual use of cough/cold medicines across countries, which would be desirable to answer whether countries truly differ in the consumption of these drugs. Similarly, differences in the working environment among responders may explain why countries with a major representation of PCPs or family medicine (Germany, Italy, Spain, and Switzerland) tend to rank with a lower score compared to a total average on some “hospital topics” such as antibiotics in newborns.

The structure of pediatric health care differs across Europe, particularly in primary care. The UK, the Netherlands, and Scandinavian countries have structured the first-line care to be delivered by general practitioners/family doctors. In contrast, in countries in Southern and Eastern Europe PCPs provide first-line curative and preventive health care for children, and some countries have mixed healthcare systems for primary care (17). In Japan, PCPs play the most important role for children; however, other specialists including internists, otolaryngologists, and dermatologists also contribute to children's medical care because of the national universal health insurance system, which allows them easy access to any specialists. The respondent characteristics in our survey reflect some of these differences and are likely to partly explain the country-specific findings. It makes sense that factors related to system structure influence the perception of medical overactivity, but further studies are needed to better identify how different healthcare systems influence specific medical overactivity.

### Strengths and limitations

To the best of our knowledge, this type of survey regarding medical overactivity with data from many countries has not been published previously. Participation from two continents and >30 countries with big differences in health systems makes our findings generalizable to a wider setting. In line with our observations, pediatricians in a similar study with participants from US hospitals ranked antibiotic overuse as three out of five top priorities (3).

We recruited participants by email, social media, different platforms, and survey announcements in meetings. Consequently, we are not able to estimate how many pediatricians became aware of the survey. Therefore, a crude response rate, cannot be explored. A potential weakness is the self-selection among participants in the survey, and we acknowledge that the sample is unlikely to be fully representative. We also noted that 20%–25% of those starting to answer the survey ended without completing their responses, and thus could not be analysed. The responses would likely be somewhat different without this self-selection, but it is not obvious how this could have influenced the responses.

This survey points to important areas for improved quality of care and initiatives against overtreatment. Recommendations and “top five lists” are among the important tools to change practice, but practice change requires multi-pronged approaches to succeed (4). Successful initiatives to reduce antibiotic overuse serve as examples of how overtreatment can be reduced by sustained concerted actions from clinicians, guideline authors, and authorities together with the collaboration of health-competent caretakers.

To conclude, in this multi-national survey, we found that overuse of supportive therapies in airway infections and antibiotics were perceived as the main challenges across more than 30 participating countries. The similarities across countries call for sharing resources and collaborating towards achieving high-quality care in pediatrics.

## Data Availability

The raw data supporting the conclusions of this article will be made available by the authors, without undue reservation.
